# Effect of Lipid Composition on the Atheroprotective Properties of HDL-Mimicking Micelles

**DOI:** 10.3390/pharmaceutics14081570

**Published:** 2022-07-28

**Authors:** Kristen Hong, Minzhi Yu, Julia Crowther, Ling Mei, Karl Olsen, Yonghong Luo, Yuqing Eugene Chen, Yanhong Guo, Anna Schwendeman

**Affiliations:** 1 Department of Pharmaceutical Sciences and the Biointerfaces Institute, University of Michigan, Ann Arbor, MI 48109, USA; kwhong@umich.edu (K.H.); minzhiyu@umich.edu (M.Y.); jcrowthe@umich.edu (J.C.); lingme@umich.edu (L.M.); olsenk@umich.edu (K.O.); 2 Department of Internal Medicine, Frankel Cardiovascular Center, University of Michigan, Ann Arbor, MI 48109, USA; yonghonl@med.umich.edu (Y.L.); echenum@med.umich.edu (Y.E.C.)

**Keywords:** micelle, atherosclerosis, high-density lipoproteins, nanoparticle, lipid composition

## Abstract

Atherosclerosis progression is driven by an imbalance of cholesterol and unresolved local inflammation in the arteries. The administration of recombinant apolipoprotein A-I (ApoA-I)-based high-density lipoprotein (HDL) nanoparticles has been used to reduce the size of atheroma and rescue inflammatory response in clinical studies. Because of the difficulty in producing large quantities of recombinant ApoA-I, here, we describe the preparation of phospholipid-based, ApoA-I-free micelles that structurally and functionally resemble HDL nanoparticles. Micelles were prepared using various phosphatidylcholine (PC) lipids combined with 1,2-distearoyl-*sn*-glycero-3-phosphoethanolamine-N-[azido(polyethylene glycol)-2000] (DSPE-PEG2k) to form nanoparticles of 15–30 nm in diameter. The impacts of PC composition and PEGylation on the anti-inflammatory activity, cholesterol efflux capacity, and cholesterol crystal dissolution potential of micelles were investigated in vitro. The effects of micelle composition on pharmacokinetics and cholesterol mobilization ability were evaluated in vivo in Sprague Dawley rats. The study shows that the composition of HDL-mimicking micelles impacts their overall atheroprotective properties and supports further investigation of micelles as a therapeutic for the treatment of atherosclerosis.

## 1. Introduction

Atherosclerosis is a main pathologic process that causes atherosclerotic cardiovascular diseases (ASCVD), including coronary heart disease (CHD), stroke and peripheral vascular disease [1]. Atherosclerotic plaques, made up of cholesterol, phospholipids, inflammatory cells, and calcium deposition, lead to the narrowing of the arteries and cause limitations of blood flow to vital organs and tissues in the body [1,2,3]. Although statins and other cholesterol-lowering drugs have been demonstrated as the most effective intervention to reduce mortality and cardiovascular events in patients with established ASCVD, statin therapy only shows a 34% decrease in the risk of major coronary events [1,4,5].

Dysregulated cholesterol metabolism and unresolved endothelial inflammation are pivotal pathogenic factors for atherosclerosis. In the early stages of atherosclerosis, the arterial endothelium gets activated by low-density lipoprotein cholesterol (LDL-C), and macrophages are recruited to the activated endothelium [6]. Phagocytizing LDL-C causes excessive intracellular cholesterol deposition in macrophages. The cholesterol-laden macrophages are converted into foam cells, causing unresolved inflammation on the artery walls through pro-inflammatory cytokines [7]. Crystallized cholesterol, which resides both intracellularly and extracellularly, also plays a detrimental role by inducing inflammation and destabilizing plaques [8,9,10]. Promoting reverse cholesterol transport, removing cholesterol crystals, and resolving endothelial inflammation would be promising treatment strategies for atherosclerosis.

In the past decade, synthetic high-density lipoprotein (sHDL) has been one of the most promising drug candidates in enhancing cholesterol efflux and resolving vascular inflammation. Typically composed of phospholipids and apolipoprotein A-I (ApoA-I) or its mimetics, sHDL mimics functions of endogenous HDLs, including mediating cholesterol efflux and resolving endothelial inflammation [11]. sHDL candidates such as CER-001 and CSL112 have entered clinical trials, where a significantly increased cholesterol efflux was observed following sHDL infusion [12,13]. In addition to sHDLs, other HDL-mimetic nanoparticles such as ApoA-I coated PLGA particles and ApoA-I functionalized gold nanoparticles are also in preclinical development [14,15]. However, the technical difficulties in the production and purification of ApoA-I have made the bench-to-bedside transition of sHDL and other HDL mimetics particularly challenging and costly [16,17,18,19,20].

Protein-free, phospholipid-based nanoparticles have long been suggested as potential anti-atherosclerotic agents due to their ability to facilitate cholesterol efflux and reduce plaque burden in animal models [21,22]. Previous research in our lab has found that a series of micelles, which are phospholipid-based nanoparticles composed of phosphatidylcholine and a pegylated phosphatidylethanolamine, showed cholesterol mobilization and plaque reduction capacities in atherosclerosis animal models with no induction of anti-PEG antibodies after IV injection [23]. To further understand the composition–activity relationship of micelles, in the present study, a series of micelles composed of different phospholipid compositions and PEGylation extents were prepared. The cholesterol crystal dissolution capacity, cholesterol efflux capacity, anti-inflammatory effects, as well as the in vivo PK/PD profiles of the micelles were evaluated, based on which the structure-activity relationship was analyzed.

## 2. Materials and Methods

### 2.1. Material

The compounds 1-palmitoyl-2-oleoyl-glycero-3phosphocholine (POPC), 1,2-dimyristoyl-*sn*-glycero-3-phosphocholine (DMPC), 1,2-dipalmitoyl-*sn*-glycero-3-phosphocholine (DPPC) and 1,2-distearoyl-*sn*-glycero-3-phosphocholine (DSPC) were purchased from NOF corporation (White Plains, NY, USA). The compound 1,2-distearoyl-*sn*-glycero-3-phosphoethanolamine-N-[azido(polyethylene glycol)-2000] (DSPE-PEG2k) was purchased from Avanti Polar Lipids (Alabaster, AL, USA). Lipopolysaccharide (LPS) (*E. coli* O111:B4) was purchased from Sigma Aldrich (St. Louis, MO, USA). Cholesterol was purchased from Sigma Aldrich (Milwaukee, WI, USA). Wako Cholesterol E Kit was purchased from Fujifilm (Richmond, VA, USA). IL-6 and TNF-α ELISA kits were purchased from Invitrogen (Ann Arbor, MI, USA).

### 2.2. Preparation of Micelle Library

All micelles were prepared using the co-lyophilization method as described previously [23,24,25]. Phosphatidylcholine (PC) lipids (POPC, DMPC, DPPC, or DSPC) and DSPE-PEG2k were dissolved in acetic acid and mixed at specified molar ratios as shown in Table 1. The resulting mixture was flash-frozen in liquid nitrogen and lyophilized overnight to remove the organic solvent. The lyophilized powder was then rehydrated with phosphate-buffered saline (PBS, pH 7.4)) and heated above and cooled below the transition temperature of each phospholipid for 10 min. This thermocycle process was repeated three times. All micelle concentrations are expressed in terms of total lipid concentration. The final micelle lipid concentration was 20 mM after preparation.

### 2.3. Characterization of Micelles

The particle size of micelles was determined using dynamic light scattering (DLS) on Malvern Zetasizer Nano ZSP (Westborough, MA, USA) at a concentration of 2 mM in PBS. Transmission electron microscopy was used to assess the morphology of micelles. At a diluted concentration of 20 μM in PBS, the samples were deposited on a carbon film-coated 400 mesh copper grid (Electron Microscopy Sciences) and negatively stained with 1% (*w*/*v*) uranyl formate. The grid was dried before TEM observation. All specimens were imaged on a 100 kV Morgagni TEM equipped with a Gatan Orius CCD.

### 2.4. Cholesterol Crystal Dissolution

Cholesterol (Sigma-Aldrich) was dissolved in pure ethanol to obtain a concentration of 2 mg/mL cholesterol solution. A total of 100 μL of cholesterol solution was transferred to each well of the 96-well plate, and then 150 μL of sterile water was added to form cholesterol crystals. After drying, cholesterol crystals were incubated with 200 µL of PBS containing indicated micelles at a concentration of 1 mM for 7 days. The supernatant was collected and cholesterol content was measured using Wako Cholesterol E Kit from Fujifilm (Richmond, VA, USA) [23].

### 2.5. Cholesterol Efflux

J774A.1 cells were cultured in DMEM supplemented with 10% FBS and 1% Penicillin-Streptomycin (10,000 U/mL). Cells were seeded in a 24-well plate at a density of 1 × 10^5^ cells/well and allowed to grow for 2 days. Cells were then labeled with 1 µCi/mL [^3^H] cholesterol (Perkin Elmer) in DMEM containing 3% fatty acid-free bovine serum albumin (BSA) (Sigma, A8806) and 5 µg/mL ACAT inhibitor Sandoz 58-035 (Sigma, S9318) and incubated overnight. The next day, cells were washed twice with PBS and equilibrated for 24 h in fresh DMEM media containing 0.3% BSA and 5 µg/mL ACAT inhibitor as described above. Cells were then incubated with DMEM containing 0.1% BSA in the presence of indicated micelles at 20 μM for 4 h at 37 °C. At the end of the incubation, the media was collected. The cells were lysed in 0.5 mL of 0.1% SDS and 0.1 N NaOH, and cell lysate was also collected. The [^3^H] cholesterol content of medium and cells was measured by liquid scintillation counting using Perkin Elmer Tri-Carb 2910TR (Waltham, MA, USA). Cholesterol efflux was presented as a percentage calculated by media counts divided by the sum of media counts and cell counts as described in previous studies [23,26].

### 2.6. Anti-Inflammatory Effects of Micelles

RAW 264.7 macrophages were obtained from ATCC and cultured in DMEM media supplemented with 10% fetal bovine serum (FBS) and 1% Penicillin-Streptomycin (10,000 U/mL) and grown in a 37 °C incubator with 5% CO_2_. Cells were seeded in a 96-well plate at 5 × 10^4^ cells/well and grown for 2–3 h. The cells were incubated with micelles (20 μM) and LPS (2 ng/mL) for 18 h. The levels of TNF-α and IL-6 pro-inflammatory cytokines in the culture media were measured using ELISA kits (Thermofisher Scientific, Ann Arbor, MI, USA).

### 2.7. Pharmacokinetic/Pharmacodynamic Evaluation in Rats

All animal experiments in the present study were approved by the Institutional Animal Care and Use Committee (IACUC) of the University of Michigan. Male Sprague-Dawley rats (7–8 weeks old) were obtained from Charles River Laboratory (Mattawan, MI, USA). Rats were randomly assigned to each treatment group, with 4 rats in each group. Rats were fasted 8 h before dosing. Rats were given PBS or different micelle formulations at 136 μmol/kg total lipid dose via tail vein injection. Blood was collected from the jugular vein in BD centrifuge tubes (BD, Franklin Lakes, NJ, USA) at predetermined time points, 0, 0.25, 1, 2, 4, 8, 24, 36, and 48 h, after dosing. Serum samples were separated by centrifugation at 10,000 rpm for 10 min at 4 °C and stored at −80 °C until further analysis [26]. At the study termination, rats were euthanized with carbon dioxide and sacrificed according to IACUC guidelines (Policy on Human Care and Use of Laboratory Animals Approved Animal Welfare Assurance Number, D16–00072 (A3114–01)).

### 2.8. Quantification of Serum Phospholipids and Cholesterol

Phospholipid, total cholesterol, and free cholesterol levels in the serum were analyzed using commercially available kits as instructed by the manufacturer (Wako Chemicals, Richmond, VA, USA). The cholesterol ester levels were calculated by subtracting the free cholesterol levels from total cholesterol levels at each time point.

### 2.9. Pharmacokinetic/Pharmacodynamic Analysis

Phoenix© WinNonlin^®^ Version 8.2 (Pharsight Corporation, Mountain View, CA, USA) was used to analyze serum concentrations of phospholipids and cholesterol vs. time profiles of each micelle formulation. A non-compartmental model was used to obtain pharmacokinetic and pharmacodynamic parameters. The pharmacokinetic parameters obtained from the plot of concentration of phospholipid versus time include the maximum plasma concentration of phospholipid (C_max_), area under the curve (AUC), elimination rate constant (K_10_), half-life of elimination (T_1/2_), total clearance of phospholipid (CL), and volume of distribution at steady state (V_ss_). The mean and coefficient of variation within each group are presented in the table. The pharmacodynamic parameters derived from total and free cholesterol, and cholesterol ester concentration versus time profiles include the area under the effect curve (AUEC), the maximum plasma concentration (E_max_) and the time at which Emax is observed (T_max_). The mean and coefficient of variation was calculated for each of the above parameters.

### 2.10. Statistical Analysis

All data are presented as mean ± SD. Significance between different micelle formulations and formulations vs. control was assessed by ordinary one-way ANOVA with Dunnett’s multiple comparisons test. Statistical difference was considered at *p* < 0.05.

## 3. Results

### 3.1. Preparation and Characterization of Micelles

**Figure 1:**
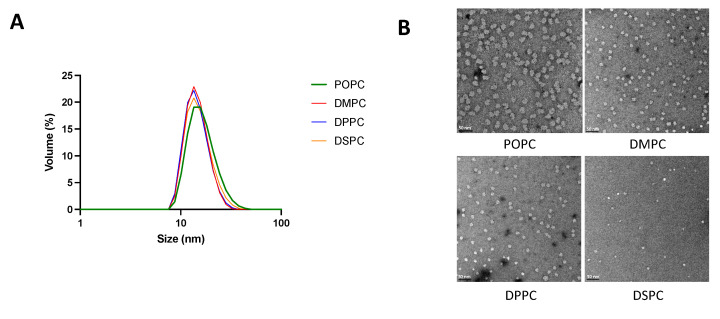
Micelle size and morphology analyzed by DLS (**A**) and TEM (**B**). Malvern Zetasizer Nano ZSP was used to determine the size of micelles diluted with PBS to a 2 mM concentration. Micelles were diluted to 20 μM in PBS and TEM images were taken on a 100 kV Morgagni TEM equipped with a Gatan Orius CCD.

Particle size and morphology of micelles composed of different phospholipids and with different PEGylation percentages were analyzed by DLS and TEM. As seen in Table 1 and Figure 1, with a fixed lipid:DSPE-PEG2k ratio of 1:2.09, micelles composed of different PC lipids all showed a uniform size distribution with an average diameter ranging from 15–18 nm. For micelles composed of DMPC and DSPE-PEG2k, on the other hand, increasing the DSPE-PEG2k percentage generally led to a reduction of particle size.

### 3.2. Cholesterol Crystal Dissolution and Cholesterol Efflux Capacity of Micelles

**Figure 2:**
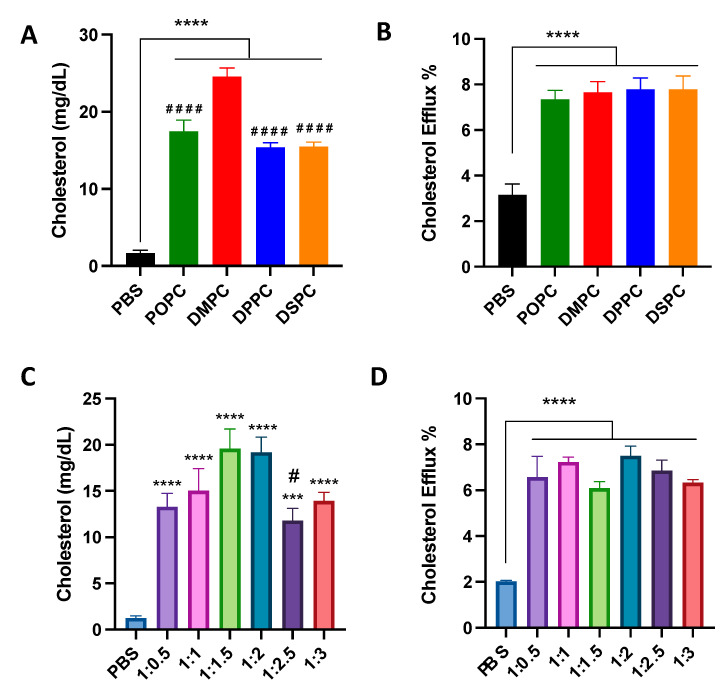
Cholesterol crystal dissolution after 7 days of incubation with different PC lipids (**A**) and different ratios of DMPC:DSPE-PEG2k (**C**). Effect of 4 h incubation of different PC lipids (**B**) and different ratios of DMPC:DSPE-PEG2k (**D**) on the cholesterol efflux of J774A.1 macrophage cells containing radiolabeled cholesterol. *** *p* < 0.001, **** *p* < 0.0001 when compared to untreated PBS group. (*n* = 3, mean ± SD. ^#^ *p* < 0.05, ^####^ *p* < 0.0001 when compared to DMPC or 1:2 group).

To examine the effect of micelle composition on cholesterol crystal dissolution capacities, micelles were incubated with cholesterol crystals at physiological temperature for 1 week. All micelles with different lipid compositions and PEGylation resulted in significant cholesterol crystal dissolution, with at least a 10-fold increase in cholesterol crystal dissolution. DMPC micelles showed more potent cholesterol crystal dissolution capacity compared to POPC, DPPC, and DSPC micelles (Figure 2A). Micelles composed of 1:1.5 and 1:2 ratios of DMPC:DSPE-PEG2k dissolved the largest concentration of cholesterol from the cholesterol crystals (Figure 2C).

Next, the cholesterol efflux capacity of micelles was tested in J774A.1 macrophages. Micelles composed of different PC lipids were able to promote cholesterol efflux, showing a 2.5 to 3-fold increase in cholesterol efflux as compared to PBS control, but there was no significant difference in the cholesterol efflux capacity of all four micelles (Figure 2B). Micelles with different ratios of DMPC:DSPE-PEG2k also showed an ability to promote cholesterol efflux, with a three-fold increase in cholesterol effluxed as compared to PBS control, though no statistical difference was observed among micelles composed of different DMPC:DSPE-PEG2k ratios (Figure 2D). Overall, the composition of micelles does not seem to substantially affect the cholesterol efflux capabilities of the nanoparticle.

### 3.3. Anti-Inflammatory Effects of Micelles

**Figure 3:**
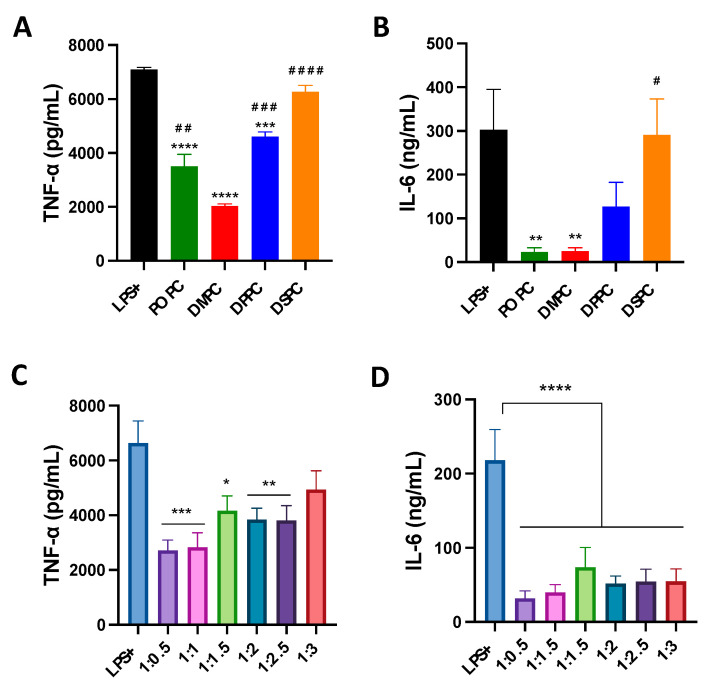
TNF-α and IL-6 pro-inflammatory cytokine release was measured after induction of inflammation with LPS endotoxin in Raw 264.7 macrophage cells with simultaneous addition of micelles composed of different PC lipids (**A**,**B**) and micelles composed of different ratios of DMPC:DSPE-PEG2k lipid (**C**,**D**). * *p* < 0.05; ** *p* < 0.01; *** *p* < 0.001: **** *p* < 0.0001 when compared to untreated LPS+ group. ^#^ *p* < 0.05; ^##^ *p* < 0.01; ^###^ *p* < 0.001: ^####^ *p* < 0.0001 when compared to DMPC group (*n* = 3, mean ± SD).

The effect of composition on the anti-inflammatory properties of micelles was evaluated in LPS-treated macrophage cells. Micelles composed of different PC lipids reduced TNF-α levels significantly compared to the LPS-only group. DMPC micelles were able to reduce TNF-α levels to the largest extent (70% reduction compared to LPS-only group), followed by POPC, DPPC and DSPC micelles (Figure 3A). A similar pattern was also observed in IL-6 levels (Figure 3B). POPC and DMPC micelles displayed a greater ability to reduce IL-6 levels than DPPC and DSPC, and showed a 90% reduction in IL-6 levels as compared to LPS-only group. As for the effects of PEGylation, micelles with less PEGylation showed stronger abilities to reduce TNF-α levels (Figure 3C). Micelles with a 1:3 DMPC:DSPE-PEG ratio performed the worst out of all formulations. At the same time, all PEGylated micelles strongly inhibited the secretion of IL-6, with a 70% reduction as compared to LPS-only group (Figure 3D). Overall, the impact of PEGylation on the anti-inflammatory activity of micelles is less significant than that of PC species.

### 3.4. Effects of PC Lipid Composition on the PK/PD Profiles

**Figure 4:**
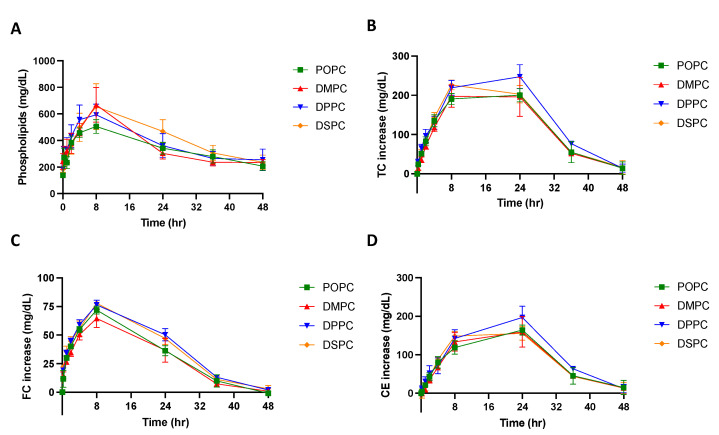
A pharmacokinetic/pharmacodynamic study was completed using Sprague Dawley Rats. Micelles were injected via tail vein injection and blood was collected from the jugular vein at various time points between 0 and 48 h post-injection. The concentration of (**A**) phospholipid, (**B**) total cholesterol (TC), (**C**) free cholesterol (FC), and (**D**) cholesterol ester (CE), over a 48-h time frame were measured using commercially available kits. Profiles of concentration vs. time are displayed over a 48 h time period (*n* = 4, mean ± SD).

Micelles composed of different PC lipids were tested in a pharmacokinetic study using Sprague Dawley rats to investigate whether PC composition affects the pharmacokinetic and pharmacodynamic parameters of the particle. The pharmacokinetic parameters were obtained by performing a non-compartmental model (NCA) analysis on the phospholipid concentration vs. time plot (Figure 4A)**.** As shown in Table 2, when comparing the micelles composed of different PC lipids, there were slight differences in the pharmacokinetic parameters. While the differences were not significant, DSPC had the largest phospholipid AUC, followed by DPPC, DMPC and POPC, suggesting that DSPC micelles had the greatest drug exposure over 48 h. Though small differences were found in the AUC and C_max_, other pharmacokinetic parameters calculated from the NCA, T_max_, CL, and V_ss_, T_1/2_ and K_10_, were all similar between micelle groups (Table 2).

To examine if the PC composition of micelles affects its ability to mobilize cholesterol in vivo, the total cholesterol (TC), free cholesterol (FC), and cholesterol ester (CE) concentrations in serum were determined and plotted on a concentration vs. time plot (Figure 4B–D). As shown in Table 3, the T_max_ of FC occurs at 8 h and the T_max_ of CE occurs at 20 or 24 h. This follows the typical pattern of response that has been seen after sHDL IV infusion, where free cholesterol is mobilized and picked up by HDL, then esterified by LCAT in the reverse cholesterol transport process, and finally eliminated through the liver. When comparing TC AUEC values, DPPC had the largest TC AUEC, followed by DSPC, POPC, and DMPC. The AUC, E_max_, and T_max_ values were not significantly different between micelle groups. All cholesterol levels returned back to baseline 48 h post-micelle injection.

### 3.5. Effects of PEGylation on the PK/PD Profiles

**Figure 5:**
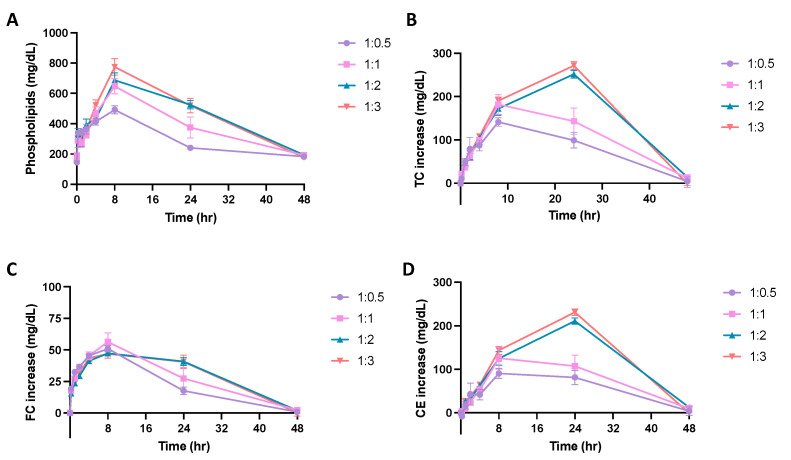
A pharmacokinetic/pharmacodynamic study was completed using Sprague Dawley Rats. Micelles were injected via tail vein injection and blood was collected from the jugular vein. The concentration of (**A**) phospholipid, (**B**) total cholesterol (TC), (**C**) free cholesterol (FC), and (**D**) cholesterol ester (CE), over a 48-h time frame were measured using commercially available kits. Profiles of concentration vs. time are displayed over a 48 h time period to examine the effect of DMPC:DSPE-PEG2k ratio on the phospholipid pharmacokinetics and cholesterol mobilization ability of the micelles (*n* = 4, mean ± SD).

To examine how the lipid ratio of DMPC:DSPE-PEG2k influences the pharmacokinetics of micelles, four out of the six different ratio micelles were chosen to test in the Sprague Dawley rats. The DMPC:DSPE-PEG2k ratio had an effect on the pharmacokinetics of micelles as seen in the phospholipid concentration vs. time plot (Figure 5A). As shown in Table 4, the C_max_ and AUC grew larger as the amount of DSPE-PEG2k increased in the micelle formulation. Micelles composed of a 1:3 ratio of DMPC:DSPE-PEG2k had a significantly larger AUC and C_max_ than 1:0.5 DMPC:DSPE-PEG2k ratio micelles, suggesting larger overall exposure and maximum serum concentration of micelles with higher PEGylation ratios. The T_1/2_ of the 1:0.5 ratio micelles was also significantly different from both 1:2 and 1:3 ratio micelles.

The ability of micelles composed of different DMPC:DSPE-PEG2k ratios to promote cholesterol mobilization was evaluated by analyzing the TC, FC and CE concentration vs. time profiles over 48 h (Figure 5B–D). Using an NCA model on WinNonlin, the T_max_, E_max_, and AUEC were determined for each cholesterol population (Table 5). As previously seen with the different PC lipid micelles, the maximum FC mobilization occurred first, followed by the peak CE mobilization, showing the subsequent elimination of cholesterol. All mobilized cholesterol was eliminated 48 h post-injection. The AUEC for TC and CE grew larger with increasing amounts of DSPE-PEG2k in the formulation, where micelles with a 1:3 ratio had a two-fold increase in AUEC as compared to the 1:0.5 micelles. For TC and CE profiles, the E_max_ was also significantly higher for 1:2 and 1:3 ratio micelles as compared to 1:0.5 ratio micelles. Overall, more cholesterol was mobilized and eliminated from the body as the amount of PEG increased in the micelles.

## 4. Discussion

The anti-atherosclerotic potential of phospholipid-based nanoparticles has long been studied due to their ability to promote cholesterol efflux, decrease inflammation, and reduce plaque burden [22,23,27]. Due to this fact, our group developed a series of phospholipid-based, HDL mimetic micelles composed of a PC lipid and DSPE-PEG2k. Recently, our group showed that a micelle composed of DPPC and DSPE-PEGk, named MiNano, was able to bind and dissolve cholesterol crystals, enhance cholesterol efflux, and suppress inflammatory responses in macrophages [23]. In this current study, we manipulate the lipid composition of micelles and we show that the lipid composition can affect micelles’ anti-inflammatory activity, cholesterol crystal dissolution abilities, cholesterol efflux capacity and in vivo PK/PD profiles.

A series of micelles composed of different phospholipids was first compared to determine the effects of PC on the therapeutic effects of micelles. PC with varying degrees of saturation and lipid tail chain lengths present different transition temperatures (T_m_) at which the lipids change phases [28,29]. Lipids with lower transition temperatures and shorter fatty acid chains, such as POPC (1-palmitoyl-2-oleoyl-glycero-3-phosphocholine) and DMPC (1,2-dimyristoyl-*sn*-glycero-3-phosphocholine), form a liquid crystalline phase at physiological temperature, where the lipids are fluid and randomly oriented. Lipids with higher transition temperatures and longer fatty acid chains and saturation, such as DPPC (1,2-dipalmitoyl-*sn*-glycero-3-phosphocholine) and DSPC (1,2-distearoyl-*sn*-glycero-3-phosphocholine), exist at a gel-ordered phase at physiological temperature, where the lipids are tightly packed and fully extended [28,30]. The phase in which the PC lipids exist may affect the ability of the lipids to interact with their molecular targets, leading to a significant pharmacological effect. Several other groups have observed differences in nanoparticle activity due to PC lipid composition changes [24,25,31,32]. Phospholipid composition has been found to significantly affect the cholesterol efflux capacity, anti-inflammatory effects, and PK/PD profiles in our previous studies on sHDL composed of ApoA-I mimetics and phospholipids. sHDLs composed of lipids with lower phase transition temperature such as DMPC and POPC have been found to have greater anti-inflammatory effects due to higher endotoxin neutralization capacity and TLR-4 displacing effects [33]. sHDL prepared with POPC and DMPC also showed a greater ability to efflux cholesterol in vitro compared to that with DPPC and DSPC [24]. However, DSPC-sHDL induced more significant cholesterol mobilization in vivo, possibly due to its longer circulation time.

Compared to previous results on peptide-containing sHDLs, there are some similarities and differences concerning the effects of phospholipids on cholesterol efflux capacities, anti-inflammatory effects, and PK/PD profiles of micelles. Similar to previous results, micelles composed of POPC and DMPC showed a greater ability to reduce pro-inflammatory cytokine levels induced by LPS (Figure 3), which may be attributed to higher LPS neutralization and/or TLR-4 displacement effects. POPC and DMPC micelles also presented better capacities in dissolving cholesterol crystals (Figure 2) as compared to micelles composed of DPPC and DSPC. However, when tested using cholesterol-loaded macrophages, no statistically significant difference was observed in cholesterol efflux among micelles composed of different phospholipids. In vivo studies suggested that changing the PC lipid of micelles did not significantly affect the PK parameters and cholesterol mobilization abilities of the micelles. Overall, the results suggested that phospholipid composition mainly affects the anti-inflammatory effects but not the cholesterol efflux capacities of micelles.

The effects of PEGylation on micelle activity and stability were also investigated. PEGylation is an extensively used strategy to extend the circulation time of nanomedicine by shielding the NPs from aggregation, phagocytosis and opsonization [26,34,35,36]. On the other hand, the shielding effects of PEG can limit the interaction between nanoparticles and target tissues, reducing the therapeutic effects of nanoparticles. In relevance to HDL-mimicking nanoparticles, Li et al. found that the addition of PEG to sHDL increased circulation time and cholesterol mobilization in rats [26]. Similarly, it was shown in this study that while micelles with different PEGylation presented comparable cholesterol efflux capacity in vitro (Figure 2), more PEGylated micelles induced a greater drug exposure and cholesterol mobilization in vivo (Table 3 and Table 4). On the other hand, PEGylation might reduce the anti-inflammatory effects of micelles (Figure 3), which could be attributed to the fact that a large amount of PEG may hinder the lipids from interacting with LPS itself or disrupt the lipid raft microenvironment which affects toll-like receptor (TLR4) recruitment. Such results suggested that the amount of PEGylation in micelles should be carefully adjusted to balance the PK profile and anti-atherogenic effects.

## 5. Conclusions

The present study highlights that the lipid composition can affect the size, anti-inflammatory activity, cholesterol crystal dissolution, cholesterol mobilization capacity and PK/PD profiles of micelles. Micelles composed of different PCs presented comparable cholesterol efflux capacity in vitro and in vivo, while micelles composed of DMPC and POPC presented more potent anti-inflammatory effects in vitro. PEGylation was found to increase the circulation time of micelles, leading to greater cholesterol mobilization when administered in vivo. The results obtained in this study may provide useful information to optimize the design of peptide-free micellar HDL mimetics for atherosclerosis therapy.

## Figures and Tables

**Table 1:** Average particle size of different micelles measured by DLS (*n* = 3, mean ± SD). Micelles were diluted to a 2 mM concentration with PBS and size was determined using Malvern Zetasizer Nano ZSP.

Formulation (Molar Ratio)	Size (nm)	PDI
POPC:DSPE-PEG2k (1:2.09).	15.97 ± 0.20	0.162 ± 0.047
DMPC:DSPE-PEG2k (1:2.09)	17.12 ± 1.09	0.298 ± 0.030
DPPC:DSPE-PEG2k (1:2.09)	18.29 ± 1.39	0.196 ± 0.028
DSPC:DSPE-PEG2k (1:2.09)	17.32 ± 0.30	0.061 ± 0.016
DMPC:DSPE-PEG2k (1:0.5)	29.74 ± 2.02	0.306 ± 0.002
DMPC:DSPE-PEG2k (1:1)	17.14 ± 0.51	0.190 ± 0.048
DMPC:DSPE-PEG2k (1:1.5)	18.88 ± 1.45	0.333 ± 0.033
DMPC:DSPE-PEG2k (1:2)	15.51 ± 0.41	0.260 ± 0.030
DMPC:DSPE-PEG2k (1:2.5)	17.69 ± 0.69	0.559 ± 0.028
DMPC:DSPE-PEG2k (1:3)	15.86 ± 0.88	0.344 ± 0.050

**Table 2:** Pharmacokinetic parameters (% CV) of phospholipids after a 136 μmol/kg dose of micelles containing different PC lipids.

Parameters	POPC	DMPC	DPPC	DSPC
**C_max_ (mg/dL)**	507.4 (20.1)	723.7 (24.1)	613.5 (25.1)	678.1 (48.5)
**T_max_ (h)**	7.0 (28.6)	7.0 (28.6)	6.7 (34.6)	7.0 (28.6)
**AUC (mg·h/dL)**	16,732.8 (21.4)	17,654.4 (18.2)	188,482.0 (32.2)	20,607.5 (37.4)
**K_10_ (h^−1^)**	0.022 (33.6)	0.027 (17.1)	0.025 (36.0)	0.026 (44.9)
**T_1/2_ (h)**	35.0 (46.5)	31.0 (41.8)	29.9 (34.5)	33.5 (81.7)
**CL (dL/h)**	0.001 (32.9)	0.001(5.4)	0.001(56.7)	0.001 (57.1)
**V_SS_ (dL)**	0.051 (20.2)	0.047 (37.9)	0.044 (20.7)	0.043 (49.4)

C_max_: the maximum plasma concentration of phospholipid; AUC: the area under the curve in a plot of concentration of phospholipid against time; K_10_: elimination rate constant; T_1/2_: the half-life of elimination; CL: total clearance for phospholipid; V_ss_: volume of distribution for phospholipid at steady state.

**Table 3:** Pharmacodynamic parameters (% CV) of total cholesterol (TC), free cholesterol (FC) and cholesterol ester (CE) after 136 μmol/kg doses of micelles containing different PC lipids.

	Parameters	POPC	DMPC	DPPC	DSPC
**TC**	**T_max_ (h)**	20.0 (40.0)	12.0 (66.7)	18.6 (49.5)	8.0 (0.0)
	**E_max_ (mg/dL)**	201.9 (16.8)	234.7 (33.6)	251.4 (20.6)	226.2 (11.0)
	**AUEC (mg·h/dL)**	6043.8 (23.9)	5925.7 (28.4)	7266.0 (16.5)	6472.5 (12.4)
**FC**	**T_max_ (h)**	8.0 (0.0)	8.0 (0.0)	8.0 (0.0)	8.0 (0.0)
	**E_max_ (mg/dL)**	72.0 (9.4)	64.5 (24.7)	76.5 (9.5)	77.9 (1.5)
	**AUEC (mg·h/dL)**	1600.0 (8.1)	1488.0 (32.1)	1923.6 (11.6)	1868.6 (9.5)
**CE**	**T_max_ (h)**	24.0 (0.0)	20.0 (4.0)	24.0 (0.0)	20.0 (4.0)
	**E_max_ (mg/dL)**	164.1 (15.9)	180.1 (37.9)	197.0 (25.9)	159.0 (18.8)
	**AUEC (mg·h/dL)**	4382.4 (30.7)	4437.7 (27.2)	5189.1 (25.3)	4492.7 (16.0)

T_max_: time at which the E_max_ is observed. E_max_: the maximum concentration of different cholesterol species. AUEC: the area under the effect curve. Data were shown as mean with CV%.

**Table 4:** Pharmacokinetic parameters (% CV) of phospholipids for micelles containing different ratios of DMPC:DSPE-PEG2k lipid dosed at 136 μmol/kg.

Parameters	1:0.5	1:1	1:2	1:3
**C_max_ (mg/dL)**	523.2 (18.0)	648.0 (15.6)	686.7 (14.2)	775.0 (14.1)
**T_max_ (h)**	6.0 (63.9)	8.0 (0.0)	8.0 (0.0)	8.0 (0.0)
**AUC (mg·h/dL)**	14,266.1 (7.0)	18,595.4 (19.7)	22,019.4 (10.1)	22,815.2 (13.5)
**K_10_ (h^−1^)**	0.023 (23.3)	0.030 (20.2)	0.032 (10.8)	0.038 (9.6)
**T_1/2_ (h)**	31.2 (20.3)	24.0 (21.3)	21.6 (10.8)	18.58 (9.3)
**CL (dL/h)**	0.006 (11.6)	0.005 (18.3)	0.005 (9.2)	0.005 (13.0)
**V_SS_ (dL)**	0.277 (12.8)	0.196 (22.7)	0.158 (13.0)	0.142 (17.4)

C_max_: the maximum plasma concentration of phospholipid; AUC: the area under the curve in plot of concentration of phospholipid against time; K_10_: elimination rate constant; T_1/2_: the half-life of elimination; CL: total clearance for phospholipid; V_ss_: volume of distribution for phospholipid at steady state.

**Table 5:** Pharmacodynamic parameters (% CV) of total cholesterol (TC), free cholesterol (FC) and cholesterol ester (CE) for DMPC micelles containing different ratios of PC:DSPE-PEG lipid dosed at 136 μmol/kg.

	Parameters	1:0.5	1:1	1:2	1:3
**TC**	**T_max,E_ (h)**	8.0 (0.0)	8.0 (0.0)	24.0 (0.0)	24.0 (0.0)
	**E_max_ (mg/dL)**	141.5 (13.7)	181.9 (25.2)	251.7 (7.2)	271.7 (6.7)
	**AUEC (mg·h/dL)**	3883.3 (17.9)	5252.1 (33.8)	7383.8 (8.7)	7813.3 (8.0)
**FC**	**T_max,E_ (h)**	8.0 (0.0)	12.0 (66.7)	8.0 (0.0)	6.0 (38.5)
	**E_max_ (mg/dL)**	50.9 (9.5)	58.4 (24.5)	47.3 (4.7)	47.3 (14.8)
	**AUEC (mg·h/dL)**	1102.7 (14.6)	1529.6 (30.0)	1511.5 (10.0)	1492.6 (23.3)
**CE**	**T_max,E_ (h)**	15.0 (70.10)	12.0 (66.7)	24.0 (0.0)	24.0 (0.0)
	**E_max_ (mg/dL)**	96.9 (19.6)	129.6 (38.1)	210.8 (6.8)	231.0 (6.6)
	**AUEC (mg·h/dL)**	2780.5 (24.9)	3722.5 (44.0)	5872.2 (10.1)	6320.7 (6.1)

T_max_: time at which the E_max_ is observed. E_max_: the maximum plasma concentration of different cholesterol species. AUEC: the area under the effect curve. Data were shown as mean with CV%.

## Data Availability

The data presented in this study are available on request from the corresponding author.
